# Use of telehealth by US adults with depression or anxiety disorder: Results from 2022 Health Information National Trends Survey

**DOI:** 10.1177/20552076251321999

**Published:** 2025-03-29

**Authors:** Pu Bai, Emily Brignone, Bibo Jiang, Casey Pinto, Li Wang

**Affiliations:** 1 Department of Public Health Sciences, 12310 Penn State College of Medicine, Hershey, PA, USA; 2 Director of Social Determinants and Research Analytics, 575429 Highmark Health, Pittsburgh, USA

**Keywords:** Telehealth, telehealth use, depression, anxiety disorder, HINTS

## Abstract

**Background:**

Telehealth use has significantly increased recently. However, little is known about its use by individuals with depression or anxiety disorders. This study aims to explore the patterns of telehealth use among those individuals.

**Methods:**

Data used were from the 2022 Health Information National Trends Survey (HINTS) cycle 6. Weighted logistic regression was performed to test the association between depression/anxiety disorder and telehealth use, and to explore reasons for using/not using telehealth among those with depression/anxiety, compared to those without.

**Results:**

Out of the 4952 study participants, 2887 (weighted percentage: 39.36%) had used telehealth in the past 12 months. Those with depression/anxiety disorder had significantly higher telehealth use, compared to those without (57% vs. 32%; OR = 2.65; 95% CI: (2.04, 3.43)). Factors affecting telehealth use could differ by depression/anxiety disorder status. Among those with depression/anxiety disorder, being woman or married was not associated with telehealth use, whereas they were significant factors among those without depression/anxiety disorder. Among those with depression/anxiety, non-Hispanic Black participants (OR = 0.51; CI: (0.78, 0.94)) were less likely to use telehealth, compared to non-Hispanic White participants; additionally, higher income was associated with telehealth use. Regarding reasons for using telehealth, convenience (OR = 1.80; CI: (1.21, 2.68)) and avoiding COVID infection (OR = 1.40; CI: (1.06, 1.86)) were more likely considered by those with depression/anxiety disorder.

**Conclusion:**

Individuals with depression/anxiety disorder were more likely to use telehealth and to do so for reasons of convenience and avoiding infection. Promoting telehealth to those with depression/anxiety disorder should consider their unique utilization patterns.

## Introduction

Depression is a major health issue in the US, with 18.5% of American adults suffering from it at some point in their lives.^1^ Depression significantly contributes to morbidity, reduced quality of life, increased economic costs and suicidal risks.^2^ Anxiety disorders are another common type of mental health condition that affects 26% of males and 40% of female respondents in the US.^3^ Treatment for depression and anxiety disorders involves two main types: medication and psychotherapy, which involve consultation and therapy sessions with mental health professionals.

Telehealth plays an increasing role in the treatment of depression and anxiety disorders. The use of telehealth exploded during the peak of COVID, when clinics reduced in-person capacity and patients avoided in-person doctors’ appointments.^4^ The use of telehealth in mental health continued after the height of COVID.^5^ In mental health, telehealth may be more widely applicable and adaptable than in physical health due to fewer physical exams/tests involved. A wide range of telehealth-based services are provided for mental health, such as individual therapy, group therapy, medication management, etc.^5^ Telehealth, as an alternative to in-person visits for care access, is especially useful for treating depression and anxiety disorder. A study found that patients with depression are more likely to use telehealth than those with schizophrenia spectrum and other psychotic disorders.^6^

Despite the benefits of utilizing telehealth for mental health,^7^ various barriers of using telehealth exist. A systematic review has identified such barriers as availability of technology, internet connectivity, patient lacking technology literacy, patient's preference for in-person visits, security/confidentiality concerns, reimbursement difficulty, etc.^8^ Mirroring the barriers are the facilitators, which counteract the barriers and promote telehealth use, such as improving technology and helping patients overcome technology difficulties.

Little is known about the patterns of telehealth use for patients with depression or anxiety disorders, although there are studies on patterns of telehealth use in overall health or mental health in general.^7–10^ It's important to understand the telehealth use patterns for depression and anxiety disorders, as they are two of the most common forms of mental health disorders. Predictors for telehealth use may be different among those with depression/anxiety disorder compared to those without, as the disease conditions may affect patients’ choice of telehealth use. The aim of this study is to identify barriers to telehealth use for individuals with depression or anxiety disorders compared to those without and to identify the reasons for their use/no-use of telehealth, using a US national survey database.

## Method

### Dataset

Our study used data from the Health Information National Trends Survey (HINTS), a nationally representative survey administered by the National Cancer Institute (NCI) on US civilian, non-institutionalized adults aged 18 or older (with or without cancer).^11^ Conducted since 2003, the HINTS surveys participants on their use of health information to manage health. This study utilized the most recent cycle data, HINTS 6, collected from March 7 – November 8, 2022.^12^ Participants for HINTS 6 were recruited through mails. The sample of mail surveys was selected from all US addresses with postal services. The survey data was collected through self-administered mail surveys plus the push-to-web mode.^13^ HINTS 6 received a total of 6252 surveys, representing a response rate of 28%.^13^ The HINTS program has provided weighting variables for research analysis that have well accounted for the non-response rate and complex survey design. Participants with missing data for variables of study interest were not included in our analysis. The public-use HINTS data and its associated analytical tools are available for free download from the Internet and require no additional permission for use. The HINTS data are validated through a rigorous development process overseen by the NCI.^14^ The complete questionnaire is available at the HINTS website. HINTS 6 was the first cycle to include telehealth section, in response to the increasing use of telehealth since COVID-19.

### Variables

#### Telehealth use

The main objective of this study is to investigate factors impacting telehealth use and to identify the reasons for using or not using telehealth. Participants were asked a multiple-choice question “In the past 12 months, did you receive care from a doctor or health professional using telehealth?” Responses of “Yes, by video” “Yes, by phone call (voice only with no video)” or “Yes, some by video and some by phone call” were considered as having used telehealth. The non-users answered, “Not using telehealth.”

#### Depression/anxiety disorder status

Our total study sample was divided into two groups by their depression/anxiety disorder status, based on participants’ answers to the question: “Has a doctor or other health professional ever told you that you had depression or an anxiety disorder?” Depending on the answer of “Yes” or “No,” participants were assigned into either the group with depression/anxiety disorder or the group without.

#### Demographics

Demographic variables included survey participants’ age, sex, race/ethnicity, marital status, occupation status, education, household income, and rural/urban status. Sex was based on the question “based on your original birth certificate, were you listed as male or female?” Occupational status was grouped into full-time work vs not, with the full-time work status defined as answering “yes” to the question “In the past 30 days, did you usually work 35 h or more per week in total at all jobs or businesses?” Urban/rural status was classified according to the urban/rural designation defined by the United States Department of Agriculture (USDA).^12^ Educational attainment level was determined by the highest grade or level of schooling the participant had completed.

#### Social determinants of health

Social determinants of health (SDOH) are a newly added category in the HINTS 6 survey. Most of the SDOH variables in HINTS 6 focus on food security and housing. One SDOH variable in HINTS, i.e., lack of transportation, could directly affect telehealth use, and was included in the analysis. The question was phrased as “In the past 12 months, how often was the following true - Lack of reliable transportation kept someone in your household from medical appointments, work, or from getting things needed for daily living?” Responses were divided into “Often true,” “Sometimes true,” and “Never true.”

#### Level of healthcare use

It is expected that the more times people receive healthcare, the more likely they will use telehealth at least once. The frequency of healthcare visits is framed in the question: “In the past 12 months, not counting times you went to an emergency room, how many times did you go to a doctor, nurse, or other health professional to get care for yourself?” Responses are grouped into “0–3 times,” “4–9 times,” and “10 or more times.”

#### Reasons for using/not using telehealth

Participants were asked the reason for using telehealth or not using telehealth. For each possible reason listed in the survey, respondents were given the option to select it or not, selecting multiple reasons allowed. Possible reasons for using telehealth included (1) avoiding COVID exposure, (2) convenience, (3) recommendation by a provider, (4) wanting to include others in the visit, (5) viewing telehealth as good as in-person visits, and (6) seeking advice for visit modality. Possible reasons for not using telehealth included (1) preference for in-person visits, (2) concerns about privacy, (3) encountering technology difficulties, and (4) not being offered the telehealth option.

### Data analysis

Weighted chi-square tests were conducted to check if variables were distributed similarly between those with depression/anxiety disorder and those without. To account for the complex sampling design, weighted analysis was used with the jackknife variance estimation. The HINTS 6 data provided a full-sample weight and a set of 50 replicate weights for variance calculation.^12^ Weighted logistic regression models were fit to examine what factors were associated with the use of telehealth, and what factors were associated with choosing a particular reason for using/not using telehealth. All statistical analyses were performed using SAS version 9.4 statistical software (SAS Institute Inc., Cary, NC, USA). The statistical significance threshold level was set at a p-value of 0.05.

## Results

Table 1 summarizes participants’ characteristics by depression/anxiety disorder status. Out of the 4952 HINTS participants included in the study, 2065 had used telehealth in the past 12 months (weighted percentage: 39.36%), and 2887 had not (weighted percentage: 60.64%). A total of 28.32% of the participants had depression/anxiety disorder. The diagnosis of depression or anxiety disorders was strongly associated with the use of telehealth (57.35% used telehealth among those with depression/anxiety disorder vs. 32.25% among those without; P < 0.0001). As shown in Table 1, these factors were associated with a higher likelihood of having depression/anxiety disorder: younger age (P = 0.0001), women (P < 0.0001), non-Hispanic white (P < 0.0001), not working fulltime (P < 0.0001), lower income (P = 0.003) and rural (P = 0.005).

**Table 1.** Characteristics of participants by depression/anxiety Status

Variables		Total Participants (N = 4952)	With Depression/Anxiety ^#^ (N = 1360; 28.32%)	Without Depression/Anxiety (N = 3592; 71.68%)
Telehealth Use (%) (p < 0.0001)	Yes (%)	39.36	57.35	32.25
	No (%)	60.64	42.65	67.75
Age category (p = 0.0001)	18–34(%)	25.83	32.72	23.10
	35–49(%)	26.87	30.38	25.49
	50–64(%)	27.71	24.75	28.88
	65–74(%)	12.32	7.67	14.16
	75+ (%)	7.28	4.48	8.38
Sex (p < 0.0001)	Male (%)	49.65	38.12	53.81
	Female (%)	50.35	60.88	46.19
Race/Ethnicity (p < 0.0001)	Non-Hispanic White (%)	62.19	71.21	58.62
	Non-Hispanic Black or African American (%)	11.04	7.87	12.29
	Hispanic (%)	16.62	12.85	18.11
	Other (%)	10.16	8.06	10.98
Marital status (p < 0.0001)	Married (%)	51.16	40.78	55.26
	Not married (%)	48.84	59.22	44.74
Educational attainment (p = 0.42)	Less than high school diploma (%)	5.77	6.07	5.64
	High school/GED or equivalent (%)	20.82	21.62	20.50
	Some college or AA degree (%)	39.54	41.12	38.92
	College or above (%)	33.87	31.19	34.93
Household Income (p = 0.0031)	Less than $20,000 (%)	13.33	20.98	10.30
	$20,000 to < $35,000 (%)	10.61	11.94	10.08
	$35,000 to < $50,000 (%)	11.76	12.27	11.56
	$50,000 to < $75,000 (%)	18.60	15.59	19.79
	$75,000 or More (%)	45.70	39.21	48.27
Work Fulltime (p < 0.0001)	Yes (%)	57.22	49.06	60.44
	No (%)	42.78	50.94	39.56
Rural or Urban (p = 0.0045)	Urban (%)	87.66	83.44	89.33
	Rural (%)	12.34	16.56	10.67
# of doctor's visits (p < 0.0001)	0–3 (%)	64.23	52.43	68.90
	4–9 (%)	26.23	32.13	23.89
	10 or more (%)	9.54	15.44	7.21
Lack Transportation (p = 0.0002)	Often (%)	3.83	9.26	1.69
	Sometimes (%)	8.81	11.35	7.81
	Never (%)	87.36	79.39	90.51
Health Insurance (p < 0.6743)	Yes (%)	89.43	90.01	89.20
	No (%)	10.57	9.99	10.80

#: All the percentages were weighted.

Table 2 shows the results from weighted multivariable logistic regression models, including various covariates. Model 1 included all study participants, model 2 only included those with depression/anxiety disorder, and model 3 was restricted to those without depression/anxiety status. In model 1, the key covariate of interest, the diagnosis of depression/anxiety disorder, was significantly associated with using telehealth (Odds Ratio – OR = 2.67; 95% CI: 2.06–3.45, p < .0001; all CIs thereafter mean 95% CI). Sex also played a significant role, with female respondents being more likely to use telehealth compared with males (OR = 1.35; CI: 1.07–1.71, p = 0.01). Married respondents were more likely to use telehealth compared with those not married (OR = 1.23; CI: 1.01–1.50, p = 0.04). The number of healthcare visits in the past 12 months was positively associated with the use of telehealth. Compared to the group with only 0–3 healthcare visits, the group with 4–9 healthcare visits in the past 12 months (OR = 2.39; CI: 1.93–2.95, p < 0.001) and the group with 10 or more times of healthcare visit (OR = 2.64; CI 1.94–3.61, p < 0.001) had higher likelihood of using telehealth. Respondents often lacking transportation to meet their daily needs were more likely to use telehealth (OR = 2.44; CI: 1.50–3.96, p = 0.0005). Respondents with health insurance coverage were significantly more likely to use telehealth (OR = 2.13; CI: 1.26–3.59, p = 0.0055). Age did not have much effect overall on telehealth use. Compared to those aged 18–34, those aged 50–74 (OR = 1.17; CI: 0.83–1.64) or 75+ (OR = 1.04; CI: 0.68–1.59) had similar likelihood of using telehealth, except for the slightly higher use by those aged 35–49 (OR = 1.51; CI: 1.08–2.11). There was little racial/ethnic effect, and multivariable analysis showed no difference in the groups of non-Hispanic Black (OR = 0.95; CI: 0.69–1.30) and Other (OR = 1.26; CI: 0.89–1.78) compared to non-Hispanic White, though a higher rate was seen in Hispanic respondents (OR = 1.48; CI: 1.07–2.03). Interestingly, Table 2 indicated that neither income level nor educational attainment was associated with telehealth use, by the multivariable analysis after controlling for other variables.

**Table 2.** Multivariable logistic regression models for telehealth use

Variable		Model 1: Total population	Model 2: With Depression/ Anxiety	Model 3: Without Depression/ Anxiety
		OR (95% CI)	OR (95% CI)	OR (95% CI)
Depression/ Anxiety disorder (Ref: No)	Yes	2.67 (2.06, 3.45)***		
Age (Ref: 18–34)	35–49	1.51 (1.08, 2.11)*	1.44 (0.83, 2.51)	1.72 (1.10, 2.69)*
	50–64	1.17 (0.83, 1.64)	1.04 (0.55, 1.96)	1.36 (0.91, 2.04)
	65–74	1.16 (0.75, 1.79)	0.94 (0.38, 2.33)	1.26 (0.76, 1.67)
	75+	1.04 (0.68, 1.59)	0.61 (0.27, 1.37)	1.27 (0.75, 2.16)
Sex (Ref: Male)	Female	1.35 (1.07, 1.71)**	1.04 (0.71, 1.52)	1.34 (1.01, 1.77)*
Race/Ethnicity (Ref: Non-Hispanic White)	Non-Hispanic Black	0.95 (0.69, 1.30)	0.51 (0.78, 0.94)*	1.13 (0.76, 1.67)
	Hispanic	1.48 (1.07, 2.03)*	1.45 (0.79, 2.67)	1.58 (1.06, 2.36)*
	Other	1.26 (0.89, 1.78)	1.33 (0.69, 2.55)	1.35 (0.89, 2.05)
Marital status (Ref: Not married)	Married	1.23 (1.01, 1.50)*	1.04 (0.71, 1.52)	1.30 (1.02, 1.65)*
Educational attainment (Ref: Less than high school)	High school graduate	1.21 (0.62, 2.34)	1.28 (0.39, 4.16)	1.39 (0.74, 2.59)
	Some college	1.28 (0.65, 2.54)	1.44 (0.47, 4.44)	1.46 (0.73, 2.92)
	College and Higher	1.65 (0.84, 3.27)	1.72 (0.54, 5.51)	1.95 (1.00, 3.77.)*
Household Income (Ref: Less than $20,000)	$20,000 to < $35,000	1.03 (0.67, 1.58)	1.62 (0.66, 3.99)	0.64 (0.37, 1.11)
	$35,000 to < $50,000	1.38 (0.67, 1.58)	2.53 (0.92, 6.94)	0.81 (0.44, 1.49)
	$50,000 to < $75,000	1.08 (0.71, 1.66)	2.40 (1.08, 5.32)*	0.62 (0.36, 1.08)
	$75,000 or More	1.47 (0.94, 2.30)	3.28 (1.53, 7.05)**	0.80 (0.46, 1.40)
Work Fulltime (Ref: No)	Yes	0.98 (0.74, 1.32)	1.03 (0.57, 1.85)	0.95 (0.72, 1.24)
Rural/Urban Area (Ref: Urban)	Rural	0.77 (0.59, 1.02)	0.64 (0.36, 1.13)	0.92 (0.66, 1.27)
No. of Healthcare Visits (Ref: 0–3)	4–9	2.39 (1.93, 2.95)***	2.86 (1.76, 4.66)***	2.30 (1.87, 2.81)***
	10 or more	2.64 (1.94, 3.61)***	3.65 (1.76, 4.66)***	2.46 (1.47, 4.14)**
Lack Transportation (Ref: Never)	Often	2.44 (1.50, 3.96)**	3.16 (1.38, 7.22)**	3.02 (1.43, 6.37)**
	Sometimes	1.22 (0.81, 1.83)	2.46 (1.10, 5.49)*	0.84 (0.52, 1.36)
Health Insurance Coverage (Ref: No)	Yes	2.13 (1.26, 3.59)**	2.25 (0.95, 5.34)	2.27 (1.36, 3.77)**

Subgroup analysis in Model 2 and 3 of Table 2 shows considerable differences by depression/anxiety disorder status when it comes to the effects of various factors on telehealth use. Factors that were significant in the group without depression/anxiety disorder could be insignificant in the other group. For example, compared with those who were 18–35, the age group 35–49 (OR = 1.44; CI: 0.83–2.51) was associated with higher telehealth use for those without depression/anxiety disorder, whereas for those with depression/anxiety, telehealth use was equally likely across all age groups. Female respondents (OR = 1.34; CI: 1.01–1.77) were more likely to use telehealth among those without depression/anxiety, but for those with depression/anxiety, both genders were equally likely to use telehealth. Married (OR = 1.30; CI: 1.02–1.65) was a significant factor among those without depression/anxiety, but not so among those with depression/anxiety. On the other hand, factors that were significant in the group with depression/anxiety disorder could become insignificant in the other group. For example, compared to non-Hispanic white participants, non-Hispanic black participants (OR = 0.51, CI: 0.78–0.94) were less likely to use telehealth among those with depression/anxiety disorder, but equally likely among those without depression/anxiety. Having income of $75,000 or more (OR = 3.28, CI: 1.53–7.05) was associated with higher likelihood of using telehealth among those with depression/anxiety but not so among those without depression/anxiety disorder.

For both the groups with and without depression/anxiety disorder, more frequent healthcare visits and a lack of transportation were both associated with the use of telehealth. Rural/urban status was not a significant factor in the adjusted analysis for both groups.

Table 3 presents the results of logistic regression models on the reasons for using telehealth, among telehealth users. The dependent variable in each model (shown in parentheses in Table 3 under each model) was the selection of the given reason for using telehealth. The models included all the covariates as those in Table 2, yet only the results on the single variable depression/anxiety disorder status were presented. Table 3 also shows the difference between those having depression/anxiety disorder vs. those who didn’t in terms of the proportion of participants selecting the given reason. Compared to those without depression/anxiety disorder, participants with depression/anxiety disorder were more likely to use telehealth for avoiding COVID-19 exposure (54.19% vs. 46.07% selected that reason; Odds Ratio:1.40; CI: 1.06–1.86; p = 0.02) and for convenience (74.29% vs. 60.79%; OR:1.80, CI: 1.21–2.68; p = 0.005). There was no significant difference in using telehealth for the reason of provider recommendation (77.43% vs. 71.43%; OR:0.25; CI: 0.85–1.86; p = 0.25). Participants with depression/anxiety disorder were equally likely to choose the reason as including family or other caregivers in their visit compared to those without (27.29% with depression vs. 20.86% without; OR:1.47; CI: 0.91–2.40; p = 0.12). Both groups were equally likely to choose the reason for using telehealth as viewing telehealth as good as in-person visits (74.60% vs. 75.48%; OR:1.18; CI: 0.79–1.76; p = 0.41). Both groups had similar rates of attributing the reason for telehealth use as seeking advice for the choice on visit modality (29.85% with depression vs. 28.71% without; OR:1.10; CI: 0.79–1.53; p = 0.58).

**Table 3.** Logistic regression models on reasons for using telehealth.

Model 1 (N = 2065) (Y: Avoiding COVID Exposure)			Model 2 (N = 2065) (Y: Convenient)			Model 3 (N = 2065) (Y: Recommended by provider)		
Proportion^#^	p	OR	Proportion^#^	p	OR	Proportion^#^	p	OR
54.19% 46.07%	0.02*	1.40 (1.06, 1.86)	74.29%; 60.79%	0.005**	1.80 (1.21, 2.68)	77.43%; 71.43%	0.25	1.25 (0.85, 1.86)
Model 4 (N = 2065) (Y: Including family in visit)			Model 5 (N = 2065) (Y: Perceiving telehealth as good as in-person)			Model 6 (N = 2065) (Y: Seeking advice for visit modality)		
Proportion^#^	p	OR	Proportion^#^	p	OR	Proportion^#^	p	OR
27.29%; 20.86%	0.12	1.47 (0.91, 2.40)	74.60%; 75.48%	0.41	1.18 (0.79, 1.76)	29.85%; 28.71%	0.58	1.10 (0.79, 1.53)

Note: “*” indicates *p *< .05. “**” indicates *p *< .01. “***” indicates *p *< .0001. OR (95% CI)

#: Proportion: The percentages in the proportion cell indicate the percentage of participants with Y = 1 (Y differs by model) among those with depression/anxiety vs. among those without depression/anxiety. E.g., 54.19% of those with depression/anxiety disorder chose “avoidance of COVID exposure” as the reason to use telehealth, whereas 46.07% without depression chose it. The list of covariates used in the logistic regression models was the same as that in the multivariable logistic regression model in Table 2.

Table 4 focuses on reasons for not using telehealth. Among the non-telehealth users, the vast majority were not offered telehealth, irrespective of depression/anxiety disorder status (78.62% of telehealth non-users with depression/anxiety disorder were not offered telehealth vs. 83.61% of those without; OR: 0.64; CI: 0.40–1.02; P = 0.06). Among those who did not use telehealth but were offered it, Model 2 through Model 4 explored if the depression/anxiety disorder status was associated with choosing a given reason as the cause of not using telehealth. Regarding the reason of preference for in-person visits over telehealth, there was no difference between those with and without depression/anxiety disorder (82.92% vs. 83.56%; OR: 0.81; CI: 0.26–2.56, p = 0.72). Privacy concerns were similarly low in both groups (12.00% for those with depression/anxiety disorder vs. 18.80% for those without; OR: 0.49; CI: 0.12–2.04; p = 0.32). Participants with depression/anxiety disorder were equally likely to cite technology difficulties as the reason for not using telehealth (14.52% vs. 19.27%; OR:0.43; CI: 0.17–1.04; p = 0.06).

**Table 4.** Logistic regression models on reasons for not using telehealth.

Model Variables	Model 1^+^ (N = 2811) (Y: Not Offered Telehealth Option)			Model 2 (N = 477) (Y: Prefer in-person visit)			Model 3 (N = 477) (Privacy concern)			Model 4 (N = 477) (Technology difficulties)		
	Prop^#^	p	OR	Prop^#^	p	OR	Prop^#^	p	OR	Prop^#^	p	OR
**Depression/Anxiety Disorder Status** **(Ref: No)**	78.62%; 83.61%	0.06	0.64 (0.40, 1.02)	82.92%; 83.56%	0.72	0.81 (0.26, 2.56)	12.00%; 18.80%	0.32	0.49 (0.12, 2.04)	14.52%; 19.27%	0.06	0.43 (0.17, 1.04)

Note: “*” indicates *p *< .05. “**” indicates *p *< .01. “***” indicates *p *< .0001.

+: Model 1 was fit among all who did not use telehealth, whereas models 2–4 were only among those who did not use telehealth AND were offered the telehealth option.

#: The percentages in the same cell indicate the percentage of participants with Y = 1 (Y differs by model) among those with depression/anxiety disorder vs. among those without depression/anxiety disorder. E.g., 78.62% of telehealth non-users with depression/anxiety were not offered the telehealth option, whereas 83.61% of those without depression/anxiety were not offered.

## Discussion

Our study focused on telehealth use for those with depression/anxiety disorder, and we found a much higher rate of telehealth use by those with depression/anxiety disorder. Factors associated with more use of telehealth could differ by depression/anxiety disorder status. The study further showed that participants with depression/anxiety disorder were more likely to attribute the reasons for telehealth use as convenience of visits and avoidance of COVID-19 exposure. The higher rate of telehealth use reflects a great potential for using telehealth to promote mental health services for patients with depression/anxiety disorder. Understanding the specific patterns and reasons for utilization is crucial for promoting telehealth to enhance access to mental health services for individuals with depression/anxiety disorder.

The high rate of telehealth use found in this study was based on data from 2022, with participants recalling telehealth use in the past 12 months, a period when the concern about COVID-19 exposure was still strong. In the current post-pandemic era, telehealth use has significantly dropped, but telehealth in mental health care remains widespread. A study published online by the Epic Research used national electronic medical record data up to the third quarter of 2023, and found that the percentage of telehealth-based encounter records for mental health increased from 7.2% in the 1st quarter of 2020 to 65.5% in the 2^nd^ quarter of 2020 during the peak of COVID. Then it gradually dropped to 41.9% in the third quarter of 2022, but remained still relatively high at 36.8% as recent as the third quarter of 2023.^15^ In contrast to mental health, telehealth use in various physical health conditions all dropped to under 11% by the third quarter of 2022, despite the previous high rates of telehealth use for physical health specialties during the 2^nd^ quarter of 2020.^15^

Demographic characteristics such as gender, age, marital status, and race/ethnicity play important roles in telehealth utilization, yet their effects may differ by depression/anxiety disorder status. Female respondents were found to be more likely to use telehealth, which is consistent with other studies that also show a higher utilization of telehealth by women.^16–18^ However, when the analysis was restricted to only those with depression/anxiety disorder in our study, both men and women were equally likely to use telehealth. This may be due to depression-associated social withdrawal tendencies,^19^ which may make telehealth more appealing among people with depression/anxiety irrespective of gender. Younger age was associated with a higher likelihood of using telehealth, but only for those without depression/anxiety. Regarding marital status, literature has consistently found its positive effect on health and health behaviors.^20,21^ The more use of telehealth by married people may be related to the influence of the first adopter of telehealth within a marital relationship. However, the marital effect was not shown for those with depression/anxiety, probably because depression was most prevalent among the youngest group, where the marriage rate was lower. Race and ethnicity also influenced telehealth use. Our study found higher telehealth use for Hispanic individuals among the total participants (regardless of depression/anxiety disorder status), consistent with existing studies.^22^ However, among only those with depression/anxiety disorder, non-Hispanic black participants were less likely to use telehealth.

In addition to those demographic factors, socioeconomic factors such as income, insurance coverage and transportation barriers were significant in promoting telehealth utilization. Higher income contributed to the use of telehealth for those with depression/anxiety disorder, probably due to better access to the internet. In contrast, lower-income individuals may face financial constraints that limit their ability to utilize telehealth services. Insurance coverage has become a facilitator for people to use telehealth, given that both public and private insurance plans have expanded their coverage on various forms of telehealth services, especially during COVID-19.^23^ The higher use of telehealth by those with transportation barriers showed that telehealth served as a critical alternative for individuals who lacked reliable transportation. It's essential to ensure that they experience equally quality care as in-person visits for those who have to use telehealth because of transportation barriers. The number of healthcare visits was significantly associated with telehealth use for both groups with and without depression/anxiety disorder.

When it comes to reasons for using/not using telehealth, there were notable differences between those with depression/anxiety disorder and those without. Telehealth users with depression/anxiety disorder were more likely to attribute utilization reasons to convenience and avoiding COVID-19 exposure. Efforts promoting telehealth should address the higher preference for convenience and safety among those with depression/anxiety disorder. There was no difference between those two groups with regards to other reasons for using telehealth, such as doctor's recommendations, family's involvement in visit, perception of equal quality or the need for advice on visit modality.

Given the specific utilization patterns and preferences of telehealth visits among individuals with depression/anxiety disorder, healthcare systems can employ tailored approach to promote telehealth to enhance mental health services access and improve care quality for this subpopulation. For example, the higher utilization rate suggests higher acceptance of telehealth, thus physicians should feel comfortable introducing telehealth to more patients with depression/anxiety disorder. Factors promoting telehealth use should be considered in making telehealth recommendations, such as income level and transportation barriers. The reasons for using or not using telehealth among those with depression/anxiety disorders are important considerations when making telehealth recommendations. From the physician's perspective, there were considerable barriers for physicians to recommend telehealth to patients. Among those who did not use telehealth, about 80% were not offered the option by their providers. Potential barriers for clinicians to provide and adopt telehealth include a shortage of experienced faculty and insufficient curricular time.^24^

There are several limitations of this study. First, the survey data were subject to self-report bias, as all the data were patient-reported. Second, for telehealth use, the HINTS data had only a single-item measure, i.e., whether or not having used some form of telehealth in the past 12 months, without the information on the frequency of visits and what conditions patients were seen for, limiting our ability to fully characterize the patterns of telehealth use. For instance, while we found that individuals with more healthcare appointments tended to use telehealth more frequently, the proportion of telehealth visits relative to the total appointments could not be assessed. The lack of detailed telehealth use information also made it impossible to differentiate between telehealth appointments for mental health care and those for other medical needs. Future research can use other database such as insurance claims data where such information is available to explore whether patients would adopt telehealth use across all types of their health care visits, once they start to use telehealth. The third limitation was the cross-sectional nature of the study, without the possibility to track patients and explore certain questions such as whether patients would continue to use telehealth in later years once starting the use in a given year.

## Conclusion

Our study identified factors associated with telehealth use for individuals with depression/anxiety disorder and explored their reasons for using/not using telehealth, compared to the counterpart of those without depression/anxiety disorder. Our findings showed notable differences between the groups with and without depression/anxiety disorder regarding telehealth use. Some factors affecting telehealth use were shared by both groups, such as the number of total healthcare visits and transportation barriers, whereas some factors were significant only for the group with depression/anxiety disorder, such as higher income. Among telehealth users, participants with depression/anxiety disorder were more likely to consider convenience and avoidance of exposure to COVID-19 as the reasons for using telehealth. Among those who were offered the telehealth option but didn’t use telehealth, there was no difference between those with depression/anxiety disorder and those without regarding reasons for not using telehealth. Healthcare systems can use these findings to promote telehealth specifically for patients with depression/anxiety disorder by addressing their specific patterns and reasons of using telehealth.
